# First molecular identification of *Echinococcus vogeli* and *Echinococcus granulosus* (*sensu stricto*) G1 revealed in feces of domestic dogs (*Canis familiaris*) from Acre, Brazil

**DOI:** 10.1186/s13071-016-1952-0

**Published:** 2017-01-14

**Authors:** Leandro Batista das Neves, Paulo Eduardo Ferlini Teixeira, Sidnei Silva, Fernanda Bittencourt de Oliveira, Daniel Daipert Garcia, Fernanda Barbosa de Almeida, Rosângela Rodrigues-Silva, José Roberto Machado-Silva

**Affiliations:** 1 Laboratório de Helmintos Parasitos de Vertebrados, Instituto Oswaldo Cruz (IOC), Fundação Oswaldo Cruz (Fiocruz), Av. Brasil 4.365, Manguinhos, Rio de Janeiro, 21045-900 Brasil; 2 Instituto Federal do Acre, Av. Coronel Brandão 1622, Xapuri, Acre 69930-000 Brasil; 3 Laboratório de Parasitologia, Instituto Nacional de Infectologia Evandro Chagas, Fundação Oswaldo Cruz (Fiocruz), Av. Brasil 4.365, Manguinhos, Rio de Janeiro, 21045-900 Brasil; 4 Laboratório de Helmintologia Romero Lascasas Porto, Departamento de Microbiologia, Immunologia e Parasitologia, Faculdade de Ciências Médicas, Centro Biomédico, Universidade do Estado do Rio de Janeiro, Rua Prof. Manoel de Abreu 444/5 andar, Vila Isabel, Rio de Janeiro, 20511-070 Brasil

**Keywords:** *Echinococcus granulosus* (*sensu stricto*), *Echinococcus vogeli*, Echinococcosis, *Canis familiaris*, Copro-PCR, Acre

## Abstract

**Background:**

*Echinococcus granulosus* (*sensu lato*) (*s.l*.) and *Echinococcus vogeli* are causative agents of chronic zoonotic diseases such as cystic and polycystic echinococcosis, respectively. In Brazil, polycystic echinococcosis has a restricted geographical distribution in the North Region, while cystic echinococcosis is observed in the South Region. Domestic dogs (*Canis familiaris*) fed with raw viscera represent a risk factor for *E. granulosus* (*s.l*.) infection in the South Region. Although this practice is frequent, it remains unclear whether domestic dogs are infected with *E. vogeli* in the state of Acre, located in the Amazon basin in the North Region of Brazil. The aim of this study was to investigate this gap in the polycystic echinococcosis epidemiology.

**Methods:**

Sixty-five fecal samples were collected from the ground in five municipalities (Sena Madureira, *n* = 14; Rio Branco, *n* = 06; Bujari, *n* = 06; Xapuri, *n* = 30; and Epitaciolândia, *n* = 09) located in the state of Acre, northern Brazil. The samples were screened for parasites by copro-PCR using the *cox*1 gene associated with automated sequencing.

**Results:**

*Echinococcus vogeli* was molecularly confirmed in a sample from Sena Madureira and *E. granulosus* (*sensu* stricto) (*s.s*.) (G1) in a sample from Rio Branco.

**Conclusions:**

These findings indicate that molecular assays are useful in typing *Echinococcus* taxa from fecal samples of dogs in northern Brazil. The present study is the first molecular record of *E. vogeli* in domestic dogs found in the state of Acre, reinforcing their role as a source of infection for humans. Because *E. granulosus* (*s.s*.) (G1) was detected for the first time in the North Region, from the epidemiological standpoint this finding is highly relevant, because it expands the known geographical distribution, which was previously restricted to the South Region of Brazil.

## Background

The domestication of canids has evolved into phenotypic changes, docility and adaptation to human-dominated environments, which has led to a lifestyle integrated into modern human societies [1]. During domestication, successful cooperation was established between humans and dogs, resulting in a variety of practical functions [2]. Thus, domestic dogs (*Canis familiaris*) fulfill various specialized activities, such as companionship, herding and hunting [3]. Nevertheless, canids have close contact with natural ecosystems and the frequent flow to peri-urban and urban habitats results in the transmission of wildlife helminthic zoonoses, often through close contact with contaminated fecal environments [4].

For example, echinococcosis is a neglected chronic zoonotic disease caused by *Echinococcus* spp. often grouped based on morphological similarities of their larval stages (metacestodes), as well as molecular-based analyses (metacestode and adult tapeworms) [5, 6]. *Echinococcus* spp. are trophically transmitted between carnivores that harbor adult tapeworms in the small intestine (definitive hosts) and livestock and rodents, where metacestodes develop in internal organs such as the liver and lungs (intermediate hosts) [7, 8]. Wild and domestic canids play a pivotal role in egg transmission to humans [7].

The geographical distribution of *Echinococcus* spp. is largely dependent on the presence of competent hosts to transmit each of them. *Echinococcus granulosus* (*sensu lato*) (*s.l*.) causes cystic echinococcosis (CE) and has a wide geographical distribution in Southern Cone countries of South America [9], affecting areas where people raise livestock in close contact with domestic/shepherd dogs [8]. In Brazil, *E. granulosus* (*s.l*.) is maintained by the dog-sheep, where herding dogs are often fed with raw livestock offal [10] in the South Region [11]. Indeed, *E. granulosus* (*sensu stricto*) (*s*.s.) (G1) (ovine strain) is the most common among canids and humans in South America [12]. *Echinococcus vogeli* causes polycystic echinococcosis (PE). It is restricted to the Neotropical Region and affects areas where people hunt pacas (*Cuniculus paca*) and live in close contact with domestic/hunting dogs [13–17]. The *E. vogeli* sylvatic cycle is based on a specific predator–prey relationship between the bush dog (*Speothos venaticus*), an indigenous canid in South America, and pacas [14, 18]. Within tropical forests, domestic dogs often accompany their owners during hunting [19] and are fed raw viscera as a reward for their activities [20]. Despite strong indications this practice can give rise to the synantropic cycle [14], it remains unclear whether such canids become infected with *E. vogeli* in the North Region of Brazil.

Over the last four decades, molecular assays have been proposed, including *E. granulosus* (*s.l*.), mitochondrial cytochrome *c* oxidase subunit 1 (*cox*1) gene-based primers [21, 22], to obtain more precise and accurate data for a better understanding of echinococcosis transmission [23–25]. Furthermore, molecular studies with greater epidemiological coverage have suggested using the copro-PCR technique to detect *E. granulosus* (*s.l*.) in canids [26–29].

This study aimed to investigate *Echinococcus* sp. DNA in feces of dogs from the state of Acre, using copro-PCR and subsequent sequencing of the mitochondrial gene cytochrome *c* oxidase subunit 1 (*cox*1).

## Methods

### Study areas

**Fig. 1:**
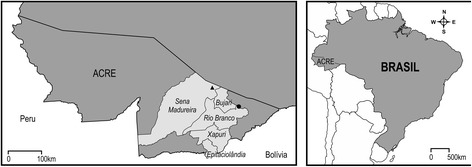
Map of Acre showing the study area and localities for *E. vogeli* (▲) and *E. granulosus* (*s.s*.) genotype 1 (●) found in feces of domestic dogs

The epidemiological survey was conducted in the state of Acre (Brazil) in August 2014 and June-July 2015 on rubber plantations and settlements in the municipalities of Rio Branco, Bujari, Xapuri and Epitaciolândia (Fig. 1). The thirty-eight rural properties that were visited in this study were located in Amazon Forest environment.

### Sample collections

Fifty-one dog fecal samples were collected in peridomestic habitats, whose residents had at least one dog. Owners were asked whether dogs engaged in hunting and/or were fed raw animal viscera, with free access to livestock viscera (cattle or sheep) and were regularly dewormed. Each fecal sample was transferred to a 50 ml tube containing 70% ethanol. The study also included 14 fecal samples from dogs living within Antimary State Forest located in the municipality of Sena Madureira. These samples were collected in 2013 and stored at the National Reference Service in Hydatidosis of the Laboratory of Helminth Parasites of Vertebrates of the IOC/Fiocruz.

### DNA extraction and polymerase chain reaction (PCR)

The macroscopic examination of tapeworm proglottids was performed. Feces were spontaneously sedimented [30] and the sediment obtained was used for molecular analysis. Total DNA extractions using the QIAamp DNA Stool Mini Kit (QIAGEN, Hilden, Germany) were realized following the manufacturer's instructions. DNA was used as template to amplify a fragment within the cytochrome *c* oxidase subunit 1 (*cox*1) mitochondrial gene as previously described [31]. The copro-PCR products were analyzed through electrophoresis in 1% agarose gel by staining with GelRed (Biotium, Hayward, USA).

### Sequence analysis

The amplicons resulting from PCR were purified using the illustra™GFX™ PCR DNA kit (GE Healthcare, Little Chalfont, United Kingdom), following the manufacturer’s instructions. Both DNA strands were sequenced using the same PCR primers and the PrimTM ABI BigDye Terminator Cycle sequencing kit (Applied Biosystems, Foster City, USA), according to the manufacturer’s instructions. The analysis of the newly-generated sequences was performed with and automated DNA sequencer (ABI 3730 analyzer, Applied Biosystems, Foster City, USA), of the RPT01A subunit for automated sequencing - IOC/Fiocruz Technology Platforms Network. The obtained nucleotide sequences were analyzed, aligned and edited by version 4.9 of the program Sequencher^TM^ (Gene Codes Corporation, USA). The nucleotide sequences obtained were aligned using the ClustalW method of the MEGA v6.0 software [32].

### Phylogenetic and distance analyses

The similarity of DNA sequencing samples of gene *cox*1 was carried out with BLAST program (Basic Local Alignment Search Tool; http://www.ncbi.nlm.nih.gov). Phylogenetic analyses were based on alignment obtained from ClustalW of thirteen 366-bp sequences and carried out with MEGA v6.0 software [32]. The phylogenetic tree was constructed using the Neighbor-Joining algorithm [33] with Kimura 2-parameter [34] model of nucleotide substitution, following the DNA Barcoding CBOL protocol (www.barcodeoflife.org/content/resources/standards-and-guidelines). To determine the robustness of the tree, bootstrap analysis of 1000 replicates was applied. The pairwise distances with the same nucleotide substitution model were calculated with MEGA v6.0 software [32]. The sequences used in the analysis were retrieved from the GenBank database under accession numbers: *E. granulosus* (*s.s*.) (G1) (U50464 [35], GU980906 [36], KC660075 [37]); *E. granulosus* (*s.s*.) (G2) (M84662 [31]); *E. granulosus* (*s.s*.) (G3) (M84663 [31], EU178105, EF545563 [38]); *E. equinus* (M84664 [31]); *E. ortleppi* (M84665 [31]); and *E. vogeli* (M84670 [31], AB208546, NC009462 [39], JX315616 [18]).

## Results

Sixty-five fecal samples were collected in peridomestic habitats (Xapuri, *n* = 30; Sena Madureira, *n* = 14; Epitaciolândia, *n* = 09; Bujari, *n* = 6; Rio Branco, *n* = 6). The pacas hunting for subsistence was a common practice for at least one family member in 33 rural properties, as well as the habits of feeding domestic dogs with raw entrails of pacas. Cattle and sheep coexisted in 7 properties and dogs had access to the carcasses of livestock. The owners of all of the properties visited admitted never having given anthelmintics to their domestic dogs.

There was no finding of dogs having passed *Echinococcus* proglottids in feces. Two samples were positive in the copro-PCR analysis. The first (DOG SM-AC-BR) was from a settlement in Sena Madureira, where dogs (*n* = 3) were fed with raw viscera from hunted animals, including pacas. The second (DOG RB-AC-BR) was from a farm in Rio Branco, where dogs (*n* = 4) had access to the carcasses of livestock (cattle and sheep).

The results of the automated sequencing showed that the DNA detected in the sample from Sena Madureira (DOG SM-AC-BR) indicated 99% similarity with 3 sequences of *E. vogeli* (M84670, AB208546, JX315616) and the sample from Rio Branco (DOG RB-AC-BR) indicated 100% similarity with 17 sequences of *E. granulosus* (*s.s*.) (G1). The nucleotide sequences of *E. granulosus* (*s.s*.) (G1) and *E. vogeli* obtained in this study were deposited in the NCBI database under accession numbers KX527915 and KX527916, respectively.

**Fig. 2:**
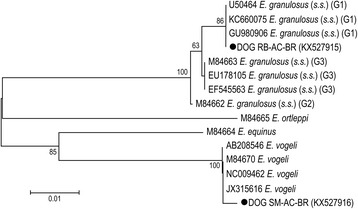
Neighbor-joining tree with Kimura 2-parameter model of substitution of the study samples and the G1–G3 genotypes of *E. granulosus* (*s.s*.), *E. equinus*, *E. ortleppi* and *E. vogeli* based on 366-bp *cox*1 sequences. The scale-bar represents a distance of 0.01 substitutions per site. Only bootstrap values above 60 are shown. Samples from this study are marked with a black circle

Phylogenetic trees based on Neighbor-Joining algorithm with Kimura 2-parameter model confirmed the species identification (Fig. 2). The sample DOG SM-AC-BR formed a cluster with *E. vogeli* and the sample DOG RB-AC-BR with *E. granulosus* (*s.s*.) (G1). The pairwise distance between our samples was 0.094 (variance 0.017). The pairwise distances between DOG SM-AC-BR and *E. vogeli* (M84670, AB208546, NC009462, JX315616) was 0.003 (variance 0.003) and between DOG RB-AC-BR and *E. granulosus* (*s.s*.) (G1) (U50464, GU980906, KC660075) was 0. Higher distances between DOG RB-AC-BR were also observed comparing this element to other sequences used for phylogenetic characterization: 0.094 (variance 0.018) for *E. ortleppi* (M84665); 0.088 (variance 0.016) for *E. equinus* (M84664); 0.008 (variance 0.005) for *E. granulosus* (*s.s*.) (G2) (M84662); and 0.006 (variance 0.004) for *E. granulosus* (*s.s*.) (G3) (M84663, EU178105, EF545563).

## Discussion

Given that dogs clearly play a critical role as working members of households (herding and hunting) in rural communities, it is important to evaluate the risk of transmission of helminthic zoonoses from dogs to humans [40]. Although humans share their environment with dogs in rural communities, this study demonstrated that owners never dewormed dogs. As a consequence, our results match the typical canine geohelminth spectrum of domestic dogs with zoonotic potential worldwide, in which fecal environmental contamination can be the main source for people in rural areas [41, 42].

In northern Brazil, polycystic echinococcosis has been reported in bush dogs [14, 18], pacas [43, 44] and humans [20, 45]. Although dogs play the double function of companion and hunting (mainly pacas) animals in these communities and are often fed raw paca viscera [14], the presence of *E. vogeli* has not yet been investigated. Over the past decade, the epidemiology of echinococcosis has achieved significant advances based on molecular assays [24, 25], including the copro-PCR technique for the detection of *E. granulosus* (*s.l*.) in canids [26–29].

Our parasitological and molecular findings give rise to some important points. First, there was no finding of dogs having passed *Echinococcus* proglottids in feces. Secondly, copro-PCR associated with automated sequencing allowed the identification of *E. vogeli* and *E. granulosus* (*s.l*.), consistent with epidemiological studies of canids in China [46] and Iran [47]. Thirdly, one *E. vogeli*-positive sample was collected from the municipality of Sena Madureira, where clinical cases and seroreactivity to polycystic echinococcosis have been described in urban and rural areas [48, 49]. Fourthly, dogs fed infected raw viscera were found to be an important risk factor in the epidemiology of echinococcosis [8, 14, 50]. In tropical forest communities, dog management practices do not include commercial food, which may be related to high poverty levels in rural communities or cultural behavior. Interestingly, owners fed raw paca meat to their dogs in Sena Madureira.

In agreement with previous investigations about *E. granulosus* (*s.l*.) [8, 12, 51], the dog-sheep cycle was confirmed, because fecal samples were collected in a farm for small ruminants, where dogs were fed offal of livestock (cattle and sheep). Historically, the Amazon Basin has been a mobile frontier of economic expansion with the spread of agropastoralism, mainly cattle ranching, from the 1970s onwards [52]. In Acre, livestock production primarily focuses on cattle but also includes sheep raising. This involved a significant migration of cattle production from other regions [50]. From the epidemiological standpoint, it is very likely that livestock movements account for the introduction of *E. granulosus* (*s.s*.) (G1) from known endemic areas in southern Brazil [10, 11, 53, 54].

To the best of our knowledge, this article contains the first report of molecularly detected *E. vogeli* infection, reinforcing the role of domestic dogs as sources of infection for humans in the municipality of Sena Madureira, where human polycystic echinococcosis has been previously reported [20, 45]. Given that *E. granulosus* (*s.s*.) (G1) was detected for the first time in the North Region, from the epidemiological standpoint this finding is highly relevant because it expands the known geographical distribution, which was previously restricted to the South of Brazil.

The strengths of our findings should be interpreted in light of limitations. In the state of Acre, during the rainy season (from October to May) the condition of unpaved roads is poor. As a consequence, field studies were only carried out in the dry season. In urban areas, sympatric domestic dogs have restricted space and movements, depending on care and food given by their owners. Management of domestic dogs in rural landscapes is difficult because they often are allowed to roam freely and the size of local dog population is unknown. Although the owners reported they owned one or more dogs, we were unable to collect feces directly after being deposited by the dogs because they were absent during our visit or defecated elsewhere.

## Conclusions

The environmental contamination with domestic dog feces is a risk factor for the transmission of helminthic zoonoses in Acre. Molecular diagnosis through copro-PCR and automated sequencing were suitable for the identification of *Echinococcus* species. To the best of our knowledge, this is the first report of *E. vogeli* and *E. granulosus* (*s.s*.) (G1) confirmed in domestic dogs from the North Region of Brazil. Further studies are needed to evaluate the extent of canine infection and the formulation of echinococcosis monitoring, considering the circulation scenario of both etiological agents.

## Abbreviations

copro-PCR: Polymerase chain reaction coprological examination
*cox*1: Cytochrome *c* oxidase subunit 1
Fiocruz: Oswaldo Cruz Foundation
G1: Genotype 1
IOC: Oswaldo Cruz Institute

## References

[1] Driscoll CA, Macdonald DW (2010). Top dogs: wolf domestication and wealth. J Biol.

[2] Range F, Virányi Z (2015). Tracking the evolutionary origins of dog-human cooperation: the “Canine Cooperation Hypothesis”. Front Psychol.

[3] Deplazes P, van Knapen F, Schweiger A, Overgaauw PA (2011). Role of pet dogs and cats in the transmission of helminthic zoonoses in Europe, with a focus on echinococcosis and toxocarosis. Vet Parasitol.

[4] Dantas-Torres F, Otranto D (2014). Dogs, cats, parasites, and humans in Brazil: opening the black box. Parasit Vectors.

[5] Alvarez Rojas CA, Romig T, Lightowlers MW. *Echinococcus granulosus sensu lato* genotypes infecting humans - review of current knowledge. Int J Parasitol. 2014;44:9–18. 10.1016/j.ijpara.2013.08.008 24269720

[6] Romig T, Ebi D, Wassermann M (2015). Taxonomy and molecular epidemiology of *Echinococcus granulosus sensu lato*. Vet Parasitol.

[7] Eckert J, Deplazes P (2004). Biological, epidemiological, and clinical aspects of echinococcosis, a zoonosis of increasing concern. Clin Microbiol Rev.

[8] Otero-Abad B, Torgerson PR. A systematic review of the epidemiology of echinococcosis in domestic and wild animals. PLoS Negl Trop Dis. 2013;7:2249. 10.1371/journal.pntd.0002249 PMC3674998 23755310

[9] Irabedra P, Ferreira C, Sayes J, Elola S, Rodríguez M, Morel N (2016). Control programme for cystic echinococcosis in Uruguay. Mem Inst Oswaldo Cruz.

[10] Farias LN, Malgor R, Cassaravilla C, Bragança C, de la Rue ML (2004). Echinococcosis in southern Brazil: efforts toward implementation of a control program in Santana do Livramento, Rio Grande do Sul. Rev Inst Med Trop Sao Paulo.

[11] Larrieu E, Zanini F (2012). Critical analysis of cystic echinococcosis control programs and praziquantel use in South America, 1974–2010. Rev Panam Salud Publica.

[12] Cucher MA, Macchiaroli N, Baldi G, Camicia F, Prada L, Maldonado L (2016). Cystic echinococcosis in South America: systematic review of species and genotypes of *Echinococcus granulosus sensu lato* in humans and natural domestic hosts. Trop Med Int Health.

[13] D’Alessandro A, Rausch RL, Morales GA, Collet S, Angel D (1981). *Echinococcus* infections in Colombian animals. Am J Trop Med Hyg.

[14] D’Alessandro A, Rausch RL (2008). New aspects of neotropical polycystic (*E. vogeli*) and unicystic (*E. oligarthrus*). Clin Microbiol Rev.

[15] Knapp J, Chirica M, Simonnet C, Grenouillet F, Bart JM, Sako Y (2009). *Echinococcus vogeli* infection in a hunter, French Guiana. Emerg Infect Dis.

[16] Vizcaychipi KA, Helou M, Dematteo K, Macchiaroli N, Cucher M, Rosenzvit M, D’Alessandro A (2013). First report of *Echinococcus vogeli* in a paca in Misiones province, Argentina. Rev Argent Microbiol.

[17] Mayor P, Baquedano LE, Sanchez E, Aramburu J, Gomez-Puerta LA, Mamani VJ, Gavidia CM (2015). Polycystic echinococcosis in Pacas, Amazon region, Peru. Emerg Infect Dis.

[18] Soares MCP, de Souza AJ S, Pinheiro Malheiros A, Nunes HM, Almeida Carneiro L, Alves MM (2014). Neotropical echinococcosis: second report of *Echinococcus vogeli* natural infection in its main definitive host, the bush dog (*Speothos venaticus*). Parasitol Int.

[19] Valsecchi J, El Bizri HR, Figueira JEC (2014). Subsistence hunting of *Cuniculus paca* in the middle of the Solimões River, Amazonas, Brazil. Braz J Biol.

[20] Siqueira NG, Siqueira CM, Rodrigues-Silva R, Soares MC, Póvoa MM (2013). Polycystic echinococcosis in the state of Acre, Brazil: contribution to patient diagnosis, treatment and prognosis. Mem Inst Oswaldo Cruz.

[21] Varcasia A, Garippa G, Scala A (2004). The diagnosis of *Echinococcus granulosus* in dogs. Parasitol.

[22] Cabrera M, Canova S, Rosenvzvit M, Guarnera E (2002). Identification of *Echinococcus granulosus* eggs. Diagn Microbiol Infect Dis.

[23] Bretagne S, Guillou JP, Morand M, Houin R (1993). Detection of *Echinococcus multilocularis* DNA in fox faeces using DNA amplification. Parasitology.

[24] Abbasi I, Branzburg A, Campos-Ponce M, Abdel Hafez SK, Raoul F, Craig PS, Hamburger J (2003). Copro-diagnosis of *Echinococcus granulosus* infection in dogs by amplification of a newly identified repeated DNA sequence. Am J Trop Med Hyg.

[25] Craig P, Mastin A, Van Kesteren F, Boufana B (2015). *Echinococcus granulosus*: Epidemiology and state-of-the-art of diagnostics in animals. Vet Parasitol.

[26] Laurimaa L, Davison J, Süld K, Plumer L, Oja R, Moks E (2015). First report of highly pathogenic *Echinococcus granulosus* genotype G1 in dogs in a European urban environment. Parasit Vectors.

[27] Al-Jawabreh A, Dumaidi K, Ereqat S, Nasereddin A, Al-Jawabreh H, Azmi K (2015). Incidence of *Echinococcus granulosus* in domestic dogs in Palestine as revealed by copro-PCR. PLoS Negl Trop Dis.

[28] Chaâbane-Banaoues R, Oudni-M’rad M, Cabaret J, M’rad S, Mezhoud H, Babba H (2015). Infection of dogs with *Echinococcus granulosus*: causes and consequences in an hyperendemic area. Parasit Vectors.

[29] Chaâbane-Banaoues R, Oudni-M’rad M, M’rad S, Mezhoud H, Babba H (2016). Environmental contamination by *Echinococcus granulosus sensu lato* eggs in relation to slaughterhouses in urban and rural areas in Tunisia. Kor J Parasitol.

[30] Lutz A (1919). O *Schistosomum mansoni* e a schistosomatose segundo observações feitas no Brasil. Mem Inst Oswaldo Cruz.

[31] Bowles J, Blair D, McManus DP (1992). Genetic variants within the genus *Echinococcus* identified by mitochondrial sequencing. Mol Biochem Parasitol.

[32] Tamura K, Stecher G, Peterson D, Filipski A, Kumar S (2013). MEGA6: Molecular Evolutionary Genetics Analysis version 6.0. Mol Biol Evol.

[33] Saitou N, Nei M (1987). The neighbor-joining method: a new method for reconstructing phylogenetic trees. Mol Biol Evol.

[34] Kimura M (1980). A simple method for estimating evolutionary rate of base substitutions through comparative studies of nucleotide sequences. J Mol Evol.

[35] Okamoto M, Bessho Y, Kamiya M, Kurosawa T, Horii T (1995). Phylogenetic relationships within *Taenia taeniaeformis* variants and other taeniid cestodes inferred from the nucleotide sequence of the cytochrome *c* oxidase subunit I gene. Parasitol Res.

[36] Soriano SV, Pierangeli NB, Pianciola L, Mazzeo M, Lazzarini LE, Saiz MS (2010). Molecular characterization of *Echinococcus* isolates indicates goats as reservoir for *Echinococcus canadensis* G6 genotype in Neuquén, Patagonia Argentina. Parasitol Int.

[37] Monteiro DU, Botton SA, Tonin AA, Azevedo MI, Graichen DA, Noal CB, de la Rue ML (2014). *Echinococcus canadensis* (G7) and *Echinococcus granulosus sensu stricto* (G1) in swine of southern Brazil. Vet Parasitol.

[38] Vural G, Baca AU, Gauci CG, Bagci O, Gicik Y, Lightowlers MW (2008). Variability in the *Echinococcus granulosus* cytochrome *c* oxidase 1 mitochondrial gene sequence from livestock in Turkey and a re-appraisal of the G1-3 genotype cluster. Vet Parasitol.

[39] Nakao M, McManus DP, Schantz PM, Craig PS, Ito A (2007). A molecular phylogeny of the genus *Echinococcus* inferred from complete mitochondrial genomes. Parasitology.

[40] Sepúlveda MA, Singer RS, Silva-Rodríguez E, Stowhas P, Pelican K. Domestic dogs in rural communities around protected areas: conservation. Problem or conflict solution? PLoS One. 2014;9:e86152. 10.1371/journal.pone.0086152 PMC3896434 24465930

[41] Varcasia A, Tanda B, Giobbe M, Solinas C, Pipia AP, Malgor R (2011). Cystic echinococcosis in Sardinia: farmers’ knowledge and dog infection in sheep farms. Vet Parasitol.

[42] Traversa D, Traversa D, di Frangipane Regalbono A, Di Cesare A, La Torre F, Drake J, Pietrobelli M (2014). Environmental contamination by canine geohelminths. Parasit Vectors.

[43] Meneghelli UG, Martinelli ALC, Velludo MASL (1990). *Echinococcus vogeli* cysts in paca liver (*Cuniculus paca*) native from the Acre State, Brazil. Rev Soc Bras Med Trop.

[44] Almeida F, Caldas R, Corrêa C, Rodrigues-Silva R, Siqueira N, Machado-Silva JR (2013). Co-infections of the cestode *Echinococcus vogeli* and the nematode *Calodium hepaticum* in the hystricomorphic rodent *Agouti paca* from a forest reserve in Acre, Brazil. J Helminthol.

[45] Meneghelli UG, Barbó ML, Magro JE, Bellucci AD, Llorach Velludo MA (1986). Polycystic hydatid disease (*Echinococcus vogeli*): clinical and radiological manifestations and treatment with albendazole of a patient from the Brazilian Amazon region. Arq Gastroenterol.

[46] Vaniscotte A, Raoul F, Poulle ML, Romig T, Dinkel A, Takahashi K (2011). Role of dog behaviour and environmental fecal contamination in transmission of *Echinococcus multilocularis* in Tibetan communities. Parasitology.

[47] Beiromvand M, Akhlaghi L, Fattahi Massom SH, Mobedi I, Meamar AR, Oormazdi H, et al. Detection of *Echinococcus multilocularis* in carnivores in Razavi Khorasan Province, Iran using mitochondrial DNA. PLoS Negl Trop Dis. 2011;5:1379. 10.1371/journal.pntd.0001379 PMC3222634 22132245

[48] Pastore R, Vitali LH, Macedo VO, Prata A (2003). A serological survey of the infection by *Echinococcus* sp. in the municipality of Sena Madureira, Acre. Rev Soc Bras Med Trop.

[49] Pastore R, Vitali LH, Weirich J, Tojal AC, Macedo VO, Prata A (2003). Hidatidosis poliquistica: relato de dois casos procedentes de Sena Madureira, Acre, na Amazônia brasileira. Rev Soc Bras Med Trop.

[50] Acosta-Jamett G, Weitzel T, Boufana B, Adones C, Bahamonde A, Abarca K (2014). Prevalence and risk factors for echinococcal infection in a rural area of northern Chile: a household-based cross-sectional study. PLoS Negl Trop Dis.

[51] Moro P, Schantz PM (2009). Echinococcosis: a review. Int J Infect Dis.

[52] McManus C, Barcellos JO, Formenton BK, Hermuche PM, Carvalho OA, Guimarães R (2016). Dynamics of cattle production in Brazil. PLoS One.

[53] de la Rue ML, Takano K, Brochado JF, Costa CV, Soares AG, Yamano K (2011). Infection of humans and animals with *Echinococcus granulosus* (G1 and G3 strains) and *E. ortleppi* in Southern Brazil. Vet Parasitol.

[54] Santos GB, Soares MCP, Brito EMF, Rodrigues AL, Siqueira NG, Gomes-Gouvêa MS (2012). Mitochondrial and nuclear sequence polymorphisms reveal geographic structuring in Amazonian populations of *Echinococcus vogeli* (Cestoda: Taeniidae). Int J Parasitol.

